# Host–Parasitoid Phenology, Distribution, and Biological Control under Climate Change

**DOI:** 10.3390/life13122290

**Published:** 2023-11-30

**Authors:** Luis Carlos Ramos Aguila, Xu Li, Komivi Senyo Akutse, Bamisope Steve Bamisile, Jessica Paola Sánchez Moreano, Zhiyang Lie, Juxiu Liu

**Affiliations:** 1 Key Laboratory of Vegetation Restoration and Management of Degraded Ecosystems, South China Botanical Garden, Chinese Academy of Sciences, Guangzhou 510650, China; lixu@scbg.ac.cn (X.L.); liezhiyang@scbg.ac.cn (Z.L.); ljxiu@scbg.ac.cn (J.L.); 2 International Centre of Insect Physiology and Ecology (*icipe*), Nairobi P.O. Box 30772-00100, Kenya; kakutse@icipe.org; 3 Unit of Environmental Sciences and Management, North-West University, Private Bag X6001, Potchefstroom 2520, South Africa; 4 Department of Entomology, South China Agricultural University, Guangzhou 510642, China; bamisopebamisile@gmail.com; 5 Grupo Traslacional en Plantas, Universidad Regional Amazónica Ikiam, Parroquia Muyuna km 7 vía Alto Tena, Tena 150150, Napo, Ecuador; jessica.sanchez@ikiam.edu.ec

**Keywords:** temperature, development, mismatch, asynchrony, altitude, warmer winters, landscape heterogeneity

## Abstract

Climate change raises a serious threat to global entomofauna—the foundation of many ecosystems—by threatening species preservation and the ecosystem services they provide. Already, changes in climate—warming—are causing (i) sharp phenological mismatches among host–parasitoid systems by reducing the window of host susceptibility, leading to early emergence of either the host or its associated parasitoid and affecting mismatched species’ fitness and abundance; (ii) shifting arthropods’ expansion range towards higher altitudes, and therefore migratory pest infestations are more likely; and (iii) reducing biological control effectiveness by natural enemies, leading to potential pest outbreaks. Here, we provided an overview of the warming consequences on biodiversity and functionality of agroecosystems, highlighting the vital role that phenology plays in ecology. Also, we discussed how phenological mismatches would affect biological control efficacy, since an accurate description of stage differentiation (metamorphosis) of a pest and its associated natural enemy is crucial in order to know the exact time of the host susceptibility/suitability or stage when the parasitoids are able to optimize their parasitization or performance. Campaigns regarding landscape structure/heterogeneity, reduction of pesticides, and modelling approaches are urgently needed in order to safeguard populations of natural enemies in a future warmer world.

## 1. Introduction

As greenhouse gas emissions increase and according to climate models, the world’s average temperature will rise by between 2.1 °C and 3.9 °C by the end of the 21st century [1,2]. Extreme heat waves, droughts, and rainfall events across regions and sectors are likely outcomes of global warming predictions [3,4], raising serious threats to global biodiversity [5,6,7,8,9,10].

Pollution, increased frequency of extreme events, as well as altered weather patterns are important drivers of insect populations [11,12,13], thus exposing them to unprecedented challenging stresses [14,15]. Increasing temperatures are the main result of global anthropogenic climate change and are disrupting interactions between herbivore–plant, predator–prey and, parasitoid–host, therefore affecting the dynamics and structure of populations and communities [16,17,18]. Additionally, the current urbanization rate and agricultural land use also threaten arthropod biodiversity and may reshape insect communities by favoring some lineages over others [19,20,21]; e.g., human landscape modification and land-use intensity (monoculture) affect host–parasitoid interactions [22] and distributions of specialist insects [23], while habitats containing patchy cropland, meadows, hedgerows, flower/grassland strips, and shelterbelts have been shown to provide greater parasitoid abundance, diversity, and parasitism rates than more simple landscape systems [24,25,26,27,28,29,30]. Furthermore, these habitats provide diverse microclimate, shelter, and structural vegetation variety that is also important for beneficial diversity and associated ecosystem services [31,32].

The presence and influence of arthropod species have significant and well-known benefits/values to human well-being in terms of the ecosystem services they provide (e.g., pollination, food security, biological control, maintenance of wider biodiversity, and ecosystem stability) and as well in achieving Sustainable Development Goals (SDGs) (e.g., crop pest and disease vectors) [33,34,35]. From the perspective of biodiversity and ecological impact, insect parasitoids are quantitatively important components of terrestrial ecosystems [36,37], because they perform a top-down control of many insect pests and consequently regulate the abundance and dynamics of their hosts [38,39,40].

**Figure 1:**
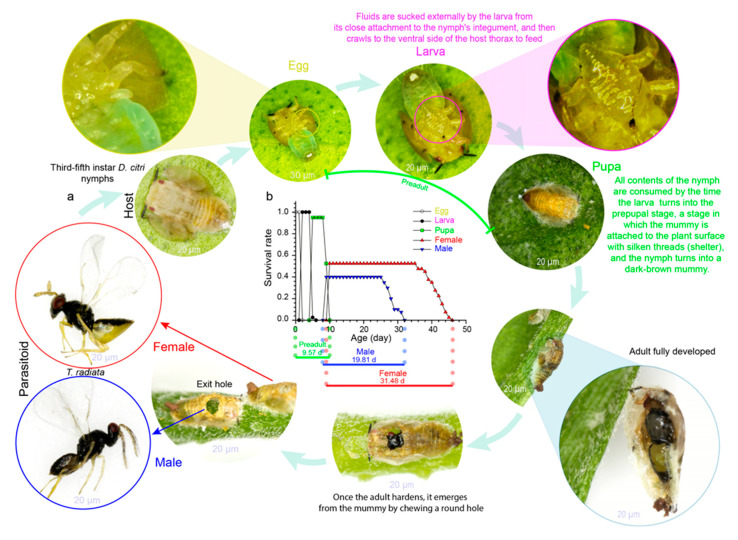
(**a**) Typical ectoparasitoid life cycle: *Tamarixia radiata* (Waterston) (Hymenoptera: Eulophidae) (credit: L.C.R.A., life cycle photos); (**b**) life-history events (phenology) of *T. radiata* reared at 27.5 °C (adapted from Ramos Aguila et al. [43]).

Regarding host–parasitoid interactions, the life cycle of insect parasitoids consists of a larval stage (parasitic) living inside the host followed by an adult stage in which the parasitoid is free-living (Figure 1a). Parasitoids depend on other insect hosts in order to develop their offspring [41]. The adult female parasitoid deposits one egg (or more than one) inside (endoparasitoid) or attached to the host surface (ectoparasitoid); the eggs hatch into larvae, which develop by feeding on their hosts’ bodies and eventually die (Figure 1a) [42].

The atmospheric temperature is intimately linked to the development and survival of parasitoid preadult/immature stages because their phenology, morphology, physiology, demography, and behavior have evolved and adapted in accordance with a specific range of thermal limits, enabling them to adapt to their surrounding environments [44]. However, it is likely that the newly predicted extreme climatic conditions will vary over time and space, thus challenging terrestrial arthropods’ life-history parameters/traits, due to the temperature-dependent nature of ectotherm activity and metabolism [45]. Nevertheless, in nature, climate changes occur over many years, decades, centuries, or longer and involve significant alterations in the averages of temperature, precipitation, wind, sunshine, etc. [46].

In this article, we organized the knowledge about climate change’s effects on parasitoid–host phenology, distribution, and biological control in light of recent publications, approaches, and advances across the disciplines that contribute to phenology research. Furthermore, we presented a link between phenological synchrony and shifts in phenology using *Diaphorina citri* (Kuwayama) (Hemiptera: Liviidae) and its associated natural enemy *Tamarixia radiata* as a model, to understanding and anticipate how climate change will impact phenology, demographics, and insect declines. Here, we also provided a review of what is known about the underlying mechanisms that govern parasitoid–host interactions in response to climate change. Additionally, we discussed approaches enabling us to draw appropriate mitigation plans and preparedness.

## 2. Arthropods’ Phenology and Climate Relationship

An organism’s phenology describes the timings of cyclical or seasonal biological events and how it progresses through its life cycle [47,48]: e.g., egg laying, the preadult developmental time (egg, larva, pupa), and adult longevity (female, male) (Figure 1b). In arthropod populations, the timing of life-history events is highly temperature-sensitive [49,50], and any change in temperature results in differential phenological shifts [51,52]. Currently, the ways in which these shifts might affect seasonal life cycles are increasingly being explored by ecologists [53]. So far, measures for climate change vulnerability have largely evaluated species’ responses to critical and lethal thermal limits [14,54]. This could be explained by its well-known direct effect on insect development [17,47], where warmer conditions accelerate preadult stages’ development [55,56]; conversely, low temperatures prolong arthropods’ developmental time [43].

Since insect metabolic rate is extremely dependent upon environmental temperature [57], any altered temperature regime is a critical factor influencing their population dynamics [58,59], mainly due to their limited capacity in maintaining body temperature through metabolic heat [60,61,62]; e.g., field experiments have demonstrated that high temperature has lethal impacts during the pupal stage releases of the parasitoid *Telenomus podisi* (Ashmead) (Hymenoptera: Platygastridae) throughout the soybean development cycle [63].

Nevertheless, in the case of parasitoids, if the ambient temperature is below the optimal temperature, increasing the temperature to close to the optimal temperature will accelerate their development [64,65,66]; however, for some species living in the tropics, the ambient temperature is near their optimal temperature (they are already living close to their thermal limits), and extreme heat waves will cause high preadult stage mortality and decrease parasitoids’ demography [44]. Furthermore, slightly warmer conditions may result in earlier adult emergence [67], benefiting some arthropod populations by increasing the number of generations per season [66], thus disrupting the relative timing of interacting species: e.g., a change in phenological synchrony between host–parasitoid interactions [5,38,68,69,70,71], affecting mismatched species’ fitness and abundance [6], disturbing ecosystem functioning [37,69,72], and ultimately leading to pest outbreaks [15,73]. For example, phenological mismatch among the cereal leaf beetle *Oulema melanopus* (Linnaeus) (Coleoptera: Chrysomelidae) and its associated parasitoid *Tetrastichus julis* (Walker) (Hymenoptera: Eulophidae) was attributed to changes in spring temperature over the years, where in warmer springs, larval phenology of *O. melanopus* was delayed relative to adult parasitoid activity and parasitism was reduced [74]. Also, increasing temperature reduces the window of the host *Agrilus planipennis’s* (Fairmaire) (Coleoptera: Buprestidae) susceptibility to *Oobius agrili* (Zhang and Huang) (Hymenoptera: Encyrtidae) parasitism [75]. In an experimental warming, development times of *Euphydryas aurinia* (Rottemburg) (Lepidoptera: Nymphalidae) were significantly affected, but not for its specialized parasitoid, *Cotesia bignellii* (Marshall) (Hymenoptera: Braconidae) [76].

Tropical ectotherms will be most adversely affected by climate change since their physiological optimum temperature is much closer to those at higher altitudes [77,78,79]. This implies that the sooner a certain degree of temperature is reached in this area, the higher the risk of extinction, since species will have less time to disperse naturally to track their physiological optimum climate. However, adaptive responses to new temperatures are also possible [80,81], since evidence of traits changing is strong; e.g., color variation of the body of the parasitoid *Cirrospilus pictus* (Nees) (Hymenoptera: Eulophidae) depends on the seasonal temperature (light individuals in spring–summer and dark individuals in autumn–winter), suggesting an ecological adaptation to climatic conditions [82]. But an explicit understanding of what underlies these changes, such as genetics or plasticity, is lacking [13]. Despite this, even within a landscape, populations and species may respond differently to climatic changes, making it difficult to identify general trends [83].

It is important to note, however, that species’ phenological shifts often do not occur at the same rate [84], and the same thermal stress can have different phenotypic and fitness effects during the various stages of an organism’s development [70,85,86]; these may consequently lead to unequal shifts in the seasonal timing [47]. For instance, recent field investigations have reported a mismatch in *Torymus sinensis* (Linnaeus) (Hymenoptera: Torymidae) emergence and a reduced biocontrol effectiveness of the Asian chestnut gall wasp *Dryocosmus kuriphilus* (Yasumatsu) (Hymenoptera: Cynipidae) as effects of warmer winter temperatures [39]. Warmer temperatures may therefore determine an earlier *T. sinensis*’s emergence, and by the time they emerge, fresh galls of the host are not available, resulting in a lower parasitism pressure and increasing the risk of host outbreaks [39]. In addition, climate-associated shifts in the phenology of wild bees have advanced by a mean of 10.4 ± 1.3 days and are associated with global temperature increases [87,88]. Also, climate change has been documented to be associated with shifts in autumn phenology toward later dates and spring phenology toward earlier dates [89,90]. Latitude has also been reported to alter the phenological responses between host and parasitoids [91], thereby affecting insect population abundance and range dynamics [55].

Insects have developed a seasonal timing system to measure day/night duration (photoperiod) and anticipate/coordinate their development and physiology [92,93]. This allows them to regulate their seasonal rhythms [94] and adapt their phenology to their local environment [52,95], in this manner allowing susceptible life stages to avoid unfavorable environmental conditions [96] and favoring the synchrony of insect populations with the resources they consume, which ultimately allows them to persist/survive [17,97]. However, new daylength regimes due to climate change are altering host–parasitoid interactions and community dynamics [98,99].

In addition, interactions within trophic networks have greatly influenced insect phenology [17]; in these interactions, organisms from a specific trophic level should regulate their life cycle to match those of their prey and hosts according to their level of trophic dependence [100]; otherwise, any phenological shifts have population-level consequences [101], therefore altering the already-established communities and systems function, and having an impact on the benefits and services provided by natural ecosystems.

## 3. Host–Parasitoid Geographical Distribution under Climate Change

**Figure 2:**
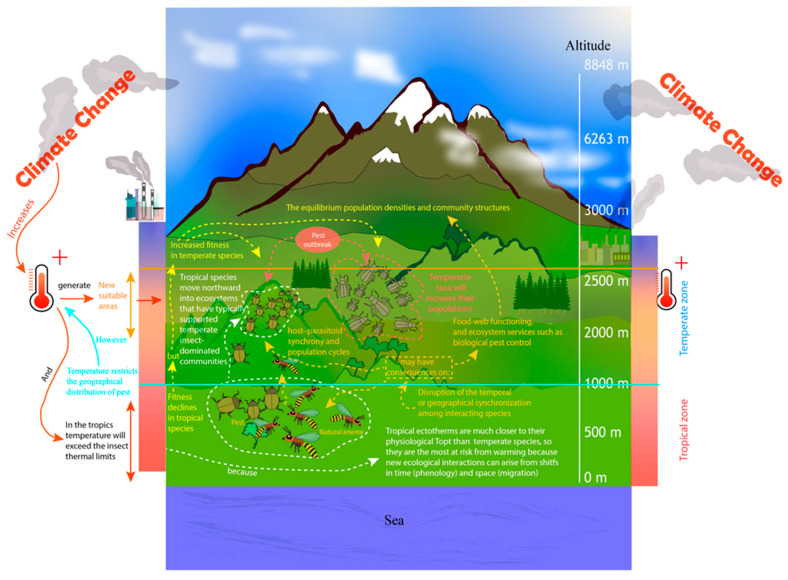
Effects of global rising temperatures on insect distribution, species interactions, and temperate taxa (created based on Johansson et al. [77]; Harvey et al. [15]; Osland et al. [107]; Parr and Bishop [79]; and Schneider et al. [105]).

A temperature limit restricts the distribution of insects; however, as a result of climate change, more suitable areas have emerged allowing species’ upslope migration (Figure 2) [66,102,103], shifting their niches to escape warming and match their current thermal preferences [52,104]. However, according to Román-Palacios and Wiens [8], niche shifts in response to climate change can only potentially reduce less than 30% of species extinction, which sparks serious concerns for the future fate of biodiversity. Agricultural pests are most likely to benefit from present and future climate change with worldwide pest proliferation, especially in temperate zones (Figure 2) [105]; e.g., warm temperatures increase population growth of a nonnative defoliator *Coleophora laricella* (Hübner) (Lepidoptera: Coleophoridae) and inhibit demographic responses of two imported parasitoids, *Agathis pumila* (Ratzeburg) (Hymenoptera: Braconidae) and *Chrysocharis laricinellae* (Ratzeburg) (Hymenoptera: Eulophidae). The positive response of hosts to warming might have contributed to the outbreak of *C. laricella* in North America [106].

Correlative species distribution modelling is a widely used approach for predicting the impacts of climate change on biodiversity, e.g., assessing extinction rates, estimating species distribution changes, and setting up conservation priorities [108,109]. Already, several insect taxa have shifted their distribution ranges towards higher altitudes [110,111]. However, regarding host–parasitoids, there is limited evidence of such geographical shifts and adaptations to these new climatic changes. For instance, *D. citri* in China has expanded significantly northward, and prediction studies revealed that this pest will move even further as a result of climate change [112]; however, using the Climate Change Experiment (CLIMEX) model, Souza et al. [113] and Aidoo et al. [114] reported that its associated natural enemy *T. radiata* will also move beyond its presently known native and non-native areas. Additionally, using climate change simulations, Li et al. [115] reported that three aphid species including *Schizaphis graminum* (Rondani)*, Rhopalosiphum padi* (Linnaeus), and *Sitobion avenae* (Fabricius) (Hemiptera: Aphididae) and their associated natural enemies *Aphidius gifuensis* (Ashmead) (Hymenoptera: Braconidae), *Episyrphus balteatus* (De Geer) (Diptera: Syrphidae), and *Harmonia axyridis* (Pallas) (Coleoptera: Coccinellidae) will move toward higher altitudes in most regions, and as the climate warms, ladybug *H. axyridis* will become more effective at suppressing aphid populations. On the contrary, warming will weaken parasitoid *A. gifuensis* and hoverfly *E. balteatus* performance and survival. Also, Zhang et al. [116] reported a northward range shift of *Anoplophora glabripennis* (Motschulsky) (Coleoptera: Cerambycidae) and its associated natural enemies *Dastarcus helophoroides* (Fairmaire) (Coleoptera: Bothrideridae) and *Dendrocopos major* (Linnaeus) (Piciformes: Picidae). According to a model studied by Furlong and Zalucki [117] on the interaction between the diamondback moth *Plutella xylostella* (Linnaeus) (Lepidoptera: Plutellidae) and its parasitoid *Diadegma semiclausum* (Hellén) (Hymenoptera: Ichneumonidae), the predicted temperature increases will negatively affect the parasitoid’s distribution more than its host’s. These studies suggested that warming can favor generalist predators over specialist (Hymenoptera) biocontrol agents.

A study carried out by Hódar et al. [118] reported that elevation decreased in both probability of occurrence and parasitism rate of the two main parasitoid species *Ooencyrtus pityocampae* (Mercet) (Hymenoptera: Encyrtidae) and *Baryscapus servadeii* (Domenichini) (Hymenoptera: Eulophidae) of the pine processionary moth *Thaumetopoea pityocampa* (Denis & Schiffermüller) (Lepidoptera: Notodontidae). Also, it is very important to consider that new host–parasitoid interactions (alternative resource species) will occur and adaptation to novel hosts is likely to increase [119,120,121,122]. In addition, with upslope range shifts, new host plants will also play a crucial role in shaping the assemblages between insect hosts and their natural enemies [123,124]. While some parasitoids are host-specific, e.g., *T. radiata* [125], others such as the case of parasitoids from the Eulophidae family (Hymenoptera: Chalcidoidea) parasitize alternative species living on wild vegetation during periods when their main host *Phyllocnistis citrella* (Stainton) (Lepidoptera: Gracillariidae) is unavailable [126]. Aphid parasitoids are also able to develop throughout the crop season on one or more host species [127]. Also, different parasitoid species can parasitize the same host; e.g., Kos et al. [128] recently reported 51 parasitoid species parasitizing the Asian chestnut gall wasp (ACGW). Other effects of global warming include (i) shifting fall migration timing in monarch butterflies *Danaus plexippus* (Linnaeus) (Lepidoptera: Nymphalidae) [129]; (ii) uphill shifts and warming altering mold body-size structures [130,131,132]; plant–pollinator mismatches [88,133]; and (iv) increasing herbivore consumption rates [134].

## 4. Warmer Winter Effects on Host–Parasitoid Interactions

As the global climate warms, fewer extreme cold events have been registered in recent decades [107], and these have generated new seasonal environment conditions (long and warmer pre-winter periods), representing a major challenge for arthropods’ life in these environments [135]. Thus, with warming, an alteration of the response to seasonal changes is expected. The survival of parasitoids within a host depends on complex physiological mechanisms, but the lethal temperature events can significantly damage these mechanisms [136,137]. This is very important to consider for interacting species because different responses to thermal performance curves (TPCs) may lead to phenological mismatches in the system [138], potentially affecting trophic interactions [139] and consequently decreasing the effectiveness/success of biological control [140]. Parasitoids, in order to be effective in regulating host pests, must have a synchronized emergence with the pest populations (i.e., suitable pest stage for parasitism), high reproduction rates, good searching/finding abilities, and a long lifespan. However, laboratory studies suggest that biological control could be negatively affected by extremes of temperature [64].

For instance, during the autumn and winter transitions [141], Senior et al. [90] reported that warmer winter temperature drives asynchronous shifts between two aphid species *Drepanosiphum platanoidis* (Schrank) (Hemiptera: Aphididae) and *Periphyllus testudinaceus* (Fernie) (Hemiptera: Aphididae), and their associated braconid parasitoid wasps (Hymenoptera: Braconidae). Similarly, the genus *Alabagrus* of braconid wasps (in the family Braconidae) and a primary parasitoid of the fern moth *Callopistaria flooridensis* (Guenée) (Lepidoptera: Noctuidae) have showed significant mismatches in emergence due to the rapid temperature increase [142]. Alford et al. [143] reported that favorable warm winters have extended the activity of the parasitoid *Aphidius avenae* (Haliday) (Hymenoptera: Braconidae), which has made them increasingly susceptible to unpredictable cold events during the winter.

**Figure 3:**
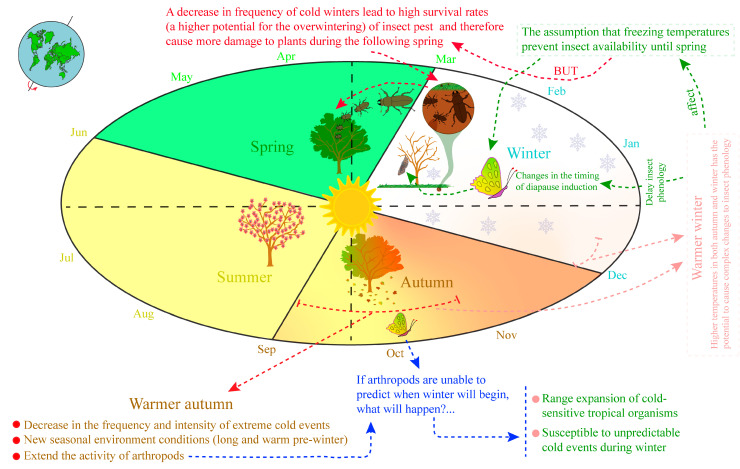
Decreases in the frequency and intensity of extreme winter cold events (long and warmer pre-winter periods) have created new seasonal environmental conditions, extended arthropod activity, allowed expansion of cold-sensitive tropical organisms, and created high pest overwintering potential. (Created based on Biella et al. [145]; Nielsen et al. [135]; and Lindestad et al. [146].)

According to Schneider et al. [144] in Switzerland, there have been fewer cold days over the past 40 years, and by the end of the 21st century, temperatures below −12 °C will occur only infrequently up to 1700 m. These events have allowed tropical cold-sensitive species to expand their ranges and colonize new areas, due to a reduction in the incidence of cold-induced physiological damage and mortality (Figure 2) [107,145]. However, for endemic arthropod species, these ambient changes represent a serious challenge, mainly because insects often enter diapause as winter approaches (Figure 3), where during this state, development stops and the metabolism is slowed/reduced, causing the body to enter a hormonally programmed resting state [146].

The aphid parasitoid *A. avenae* has been known to adopt a winter diapausing strategy, until recent reports of active winter populations in cereal crops [147]. Also, Alfaro-Tapia et al. [148] reported that diapause incidence of aphid parasitoids did not increase during winter in the Chilean central-south valley; instead, activity and abundance of parasitoids were observed. However, a study by Mehrnejad and Copland [149] on the parasitoid *Psyllaephagus pistaciae* (Ferrière) (Hymenoptera: Encyrtidae) reported that a 100% diapause was produced when low temperature was combined with a short-day photoperiod, which led to an increase in diapause incidence. A laboratory study under nine different photoperiods and temperature conditions by Tougeron et al. [150] reported that two historically winter-active parasitoid species *Aphidius rhopalosiphi* (Esenbeck) (Hymenoptera: Braconidae) and *Aphidius matricariae* (Haliday) (Hymenoptera: Braconidae) never entered diapause; in contrast, two species more recently active during winter, *A. avenae* and *Aphidius ervi* (Haliday) (Hymenoptera: Braconidae), did enter diapause but at a low proportion. Tougeron et al. [151] suggested that this recent modification in the composition of parasitoid community is linked to shifts in diapause expression (reduction of the use of winter diapause). These results suggest that aphid parasitoids’ overwintering strategies have changed rapidly in the last three decades and active adult overwintering can replace diapause; this new species will affect the food web structure between aphids and parasitoids as well as host-exploitation strategies of parasitoids already existing in the system.

Daylength and temperature are the primary factors by which diapausing insects anticipate and prepare for harsh conditions [152]. According to Polgár et al. [153], Brodeur and McNeil [154], and Polgár and Hardie [155], parasitoids also enter diapause based on host life cycle, development stage, species, size, host morph, and host plant quality. However, a question that has been less explored is what happens when organisms are unable to predict when winter will actually begin, since they need to enter diapause well before hostile conditions arrive. Any changes in diapause timing and duration generally determine or affect the number of generations per year [156]. Warmer winters may have a particularly strong effect on the biological processes of insects’ life cycles (i.e., eclosion from pupation) that are adapted to survive and overcome the winter’s coldest conditions [135,146,157].

Xiao et al. [158] reported that an increased mortality of arthropods may result from warmer winter conditions during dormant diapause, because warming conditions can reduce nutritional reserves and lead to changes in larval body weight and suffering from higher mortality. According to Wu et al. [130], a decline in the size of communities can be expected if there are widely observed reductions in the developmental size with climate warming. Indeed, Forister et al. [159] reported that in the past four decades, the number of butterflies observed has declined by 1.6% annually across landscapes of West America, and this decline was associated in particular with warmer months in the autumn. Nice et al. [83] reported that late spring precipitation as an outcome of global warming has negatively impacted butterfly populations. Dahlhoff et al. [160] also reported that in the Sierra Nevada mountains, low snowpack drives a decrease in the population abundance of the leaf beetle *Chrysomela aeneicollis* (Schaeffer) (Coleoptera: Chrysomelidae). Several pollinators, including the beetle *Mylabris nevadensis* (Escalera) (Coleoptera: Meloidae), were negatively affected by warming in Mediterranean regions [161]. Soroye et al. [162] also found that increasing frequency of unusually hot days is leading to increasing local arthropod extinction rates, reducing colonization and site occupancy and decreasing species richness within a region. Also, Burkle et al. [88] reported loss of species, co-occurrence, and function of plant–pollinator interactions over a 120-year timespan in Carlinville, Illinois (USA). It has been estimated that worldwide insect losses are approximately 9% per decade [163,164]. Currently, there is mounting evidence that arthropods are disappearing rapidly, with climate change being the main contributing factor [8,164,165,166,167,168,169,170,171]. According to Warren et al. [172], the geographic range losses of insects will reach 18% with 2 °C increases in temperature. As a result of these findings, climate change may threaten seasonal organisms in the future and may reduce insect survival over the winter, in this manner reshaping insect biodiversity worldwide [8,167,173].

## 5. Temperature Tolerance Ranges and Implications for Biocontrol Efficacy

**Figure 4:**
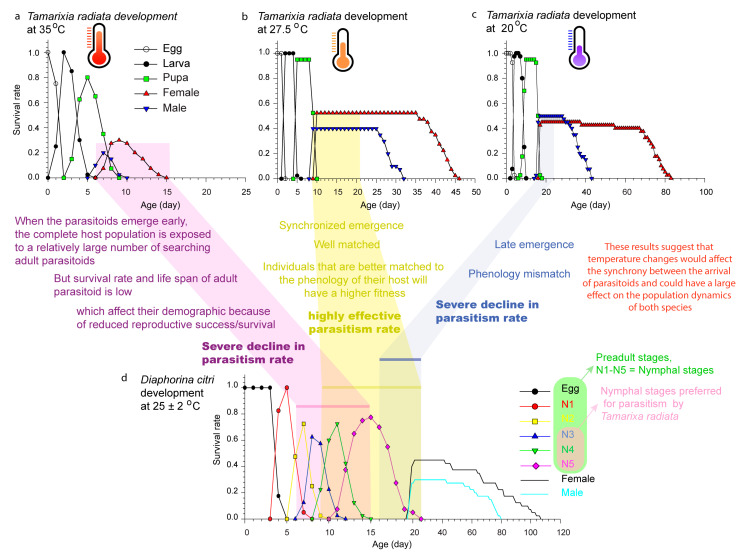
(**a**–**c**) Life-history events of *T. radiata* at 35, 27.5, and 20 °C, respectively (adapted from Ramos Aguila et al. [43]); and (**d**) life-history events of *D. citri* at 25 ± 2 °C (adapted from Ramos Aguila et al. [177]). The variation in temperature over the course of the year as a result of climate change is not uniform, and thus can easily lead to differential phenological shifts and thereby to mismatches among the interacting species.

The effects of heat stress and lower humidity (i.e., summer droughts) are detrimental to insect neurological function, muscular control, and immune function, resulting in coma and eventual death in severe situations [174,175]. TPCs have been widely used to determine and understand insect thermal plasticity and adaptation [176] and global warming effects [66]. As temperature increases, parasitoid performance typically increases proportionally, reaching its peak at optimum temperatures (Topt) (Figure 4b) [38], after which any increase in temperature produces a decline in their performance (Figure 4a) [65].

Under warming conditions, both the host and the parasitoid will develop faster, although the hosts have a higher thermal limit than their associated parasitoid [117]. Indeed, the thermal tolerance of parasitoids is lower compared to their hosts [70,178], giving them limited plasticity to respond to high temperatures and decreasing parasitoid biomass [73,117]. In a thermal study carried out by Moore et al. [179], the authors reported that the parasitoid wasp *Cotesia congregata* (Say) (Hymenoptera: Braconidae) suffered complete mortality at a temperature range that was slightly stressful for its larval host *Manduca sexta* (Linnaeus) (Lepidoptera: Sphingidae). Also, Andrade et al. [180] reported that the emergence rates of *Trichogramma exiguum* (Pinto & Platner) and *Trichogramma acacioi* (Brun, Gomez de Moraes & Soares) (Hymenoptera: Trichogrammatidae) were significantly affected at 30 °C; there was also a higher incidence of *Trichogramma* parasitism in climates with lower seasonality [181]. A very high mortality rate of the immature stages of *Aganaspis daci* (Weld) (Hymenoptera: Figitidae), a natural enemy of *Ceratitis capitata* (Wiedemann) (Diptera: Tephritidae), was observed at 15 and 30 °C [182]. Qiu et al. [183] reported that at 26 °C *Microplitis manilae* (Ashmead) (Hymenoptera: Braconidae) presented maximum parasitism rate on *Spodoptera exigua* (Hübner) and *Spodoptera litura* (Fabricius) (Lepidoptera: Noctuidae), which significantly dropped at 32 °C. Similarly, other laboratory experiments have demonstrated a reduced parasitism rate, short lifespan, and high pupal mortality when temperature exceeded the thermal limit of the parasitoids [64,179,184,185,186].

In biological control, the timing of biological activities and life-history events (i.e., stage differentiation = metamorphosis) of a pest and its associated natural enemy must be accurately described in order to determine the exact time of the host’s susceptibility or stage when the parasitoid/predator can parasitize/prey on their hosts. Life table analysis is a research tool commonly used in population and community ecology studies; this principle has been used as the basis for parasitoid–host, predator–prey studies due to its ability to graphically illustrate and describe the unique and important features of stage differentiation [64,187]. This knowledge is therefore a key component in biological control programs in achieving successful pest management. However, the stage differentiation of arthropods is temperature-dependent, and the current rising temperature due to climate change has disrupted the synchrony of host–parasitoid interaction networks. Disrupted synchronization implies that the future mass rearing of parasitoids and predatory natural enemies might face serious problems, primarily because increasing temperature accelerates arthropods’ development rate, shifts the timing of emergence, and shortens the window of host susceptibility since species of varying trophic levels respond differently to climate variations, consequently modifying the normal already known stage differentiation and developmental rate; consequently, the release of exotic parasitoids, could fall within a wrong timing (during the wrong phenological development of the target pest species), resulting in unsuccessful establishment, performance, and spread of these biocontrol agents.

An important step often omitted, and which needs great attention in successful biological control, is the link between phenological synchrony and shifts in phenology that impact population dynamics. Establishing these links is the first step to understanding and anticipating how climate change will impact phenology, demography, and insect declines. Figure 4 shows the developmental stages (egg, larva, pupa, adult) of *T. radiata*, an ectoparasitoid of *D. citri* reared at normal temperature of 27.5 °C with a mean preadult duration of 9.57 days (d) (Figure 4b) and extreme temperatures of 35 and 20 °C, with mean preadult durations of 7.29 and 16.53 d, respectively (Figure 4a,c) [43], and its host *D. citri* reared at 25 ± 2 °C, with a mean preadult duration of 18.20 d (Figure 4d) [177].

When analyzing the curves of *T. radiata* at 35 °C (Figure 4a), the authors indicate that the parasitoids’ preadult development is 2.28 and 9.24 d faster than at 27.5 and 20 °C, respectively, as well as the adult emergence, which is also observed to be faster. When projecting their biological control effectiveness on *D. citri* (Fuchsia color), *T. radiata* would match the ideal instar for parasitism. However, at this temperature (35.5 °C), the parasitoid survival rate and adult longevity is very low (Figure 4a), resulting in a severe decline in parasitism rate and reduced biological control effectiveness [64]. As a result, these two species have a mismatch in their interactions. The curves at 27.5 °C (Figure 4b) also show that adults of *T. radiata* emerge at the ideal time for parasitism when the host is in the 3–5th instar (light yellow color); parasitism, survival rate and longevity are high; and phenology does not differ. When analyzing the curves at 20 °C (Figure 4c) (light blue color), it was observed that the parasitoids take more time to emerge as adults, and by the time they emerge, it is too late; therefore, adults will parasitize only a small number of the host nymphs, since *D. citri* nymphs are then finishing the last nymphal development (N5), indicating that the two species’ interactions are thus mismatched. After analyzing these laboratory experiment results as evidence, we can see that extreme temperature regimes shifted the parasitoids’ phenology and the majority of individuals emerged earlier (or later) than the optimal time window or host susceptibility, resulting in differential phenological shifts and thereby mismatches between the interacting species.

Also, temperatures affect endosymbiont bacteria (temperature-sensitive symbiotic partners) present in parasitoids [188]; e.g., *Buchnera* and *Wolbachia*, two dominant groups of endosymbionts present in parasitoids and hosts, may be affected or eliminated if exposed to short-term high temperatures [189,190,191]. When reducing their population, this reduction is reflected in the fitness and several aspects of the parasitoids’ life-history traits [190,192], because endosymbionts act as nutritional mutualists boosting/regulating the vital functions of their host [193,194]. Furthermore, different synchronization mismatches among predators and prey as a result of raising temperature have been documented [17,195,196].

## 6. Conclusions

Recent evidence of marked host–parasitoids’ phenological shifts, geographical distribution, and reduced biological control as side effects of climate change sparked global concerns and highlighted the vital role that phenology plays in ecology due to its ecological and economic importance for ecosystem functioning. Host–parasitoid interactions are affected by the effects of global warming through a variety of mechanisms, primarily because temperature accelerates their metabolism and growth, thus affecting their biological activities and life-history events. Beyond the impacts on individual organisms, these changes are affecting the higher trophic levels, altering already-established communities and ecosystem functions. In order to gain insight into host–parasitoid populations’ reactions to altered temperature regimes, results from laboratory and field experiments must be incorporated into long-term monitoring programs. We therefore need to conduct more field studies in natural ecosystems, in order to obtain a better understanding of the effects of temperature on the host–parasitoid system and the trophic levels adjacent to them. Additionally, human-induced stresses such as farming and cow breeding intensification, introduction of exotic species, land use, pollution, habitat loss, and fragmentation are all together contributing to increasing the global temperature, and this is driving sharp phenological mismatches among host–parasitoid systems throughout the planet. To reduce climate change, agricultural practices must be redesigned in order to reduce CO_2_ emissions; in particular, a significant reduction in cow breeding and chemical pesticide inputs is needed and, in place of it, more eco-friendly and sustainable practices need to be adopted, in particular for intensively farmed areas. For example, improved landscape planning—heterogeneity and configuration—at both local and wide areas will be essential to promote parasitoid biodiversity and maintain essential ecological services, because these approaches have been shown to harbor natural enemies that are crucial to the control of herbivorous pest species that pose a threat to many crops. Therefore, there is an urgent need for these strategies to be promoted and implemented to reverse or slow down current trends and allow the recovery of parasitoid populations by providing suitable habitats for them and consequently safeguarding the vital ecosystem services they provide.

## Data Availability

No new data were created or analyzed in this study. Data sharing is not applicable to this article.
